# Medical Society of Tennessee

**Published:** 1842-08

**Authors:** 


### MEDICAL SOCIETY OF TENNESSEE.

The regular annual meeting of this society was held at Nashville on the first Monday in May, and was attended by about its average number of members. Dr. Buchanan, the Vice President, delivered the annual address, on the pathology of fever, which will be published soon among the proceedings of the Society. In the reading, it struck us as one of the most elaborate and logical papers that we have heard for a good while, and we are a good deal mistaken if it does not enhance the reputation of the author as a sound thinker and a clear and learned writer. Dr. Richardson, of Rutherford, read a practical and judicious paper on the medical topography and diseases of that county, which has been promised us for publication in the Journal. Several of the cases reported possessed practical interest, some of which we expect to publish. Upon the whole, the meeting was characterised by as much spirit as has usually marked the meetings of the Society, and as the attendance has been reduced chiefly to the members who take part in the literary exercises, and believe that the beneficial influence of the association is to be derived from that source, it is probable that it will not decline below its present state, which, if not one of youthful vigor and hope, is yet one of respectable solidity and animation.

The officers elected were, Dr. Buchanan, of Columbia, President; Dr. Geo. Thompson, of Jefferson, Vice President; Dr. R. Martin, of Nashville, Recording Secretary; Dr. Shelby, Treasurer, and Dr. Richardson, of Rutherford, Orator. The transactions of the Society are to be published shortly, when we shall be able to speak more at length of its proceedings. Y.

